# A Novel Lineage of Endosymbiotic *Actinomycetales*: Genome Reduction and Acquisition of New Functions in *Bifidobacteriaceae* Associated With Termite Gut Flagellates

**DOI:** 10.1111/1462-2920.70010

**Published:** 2025-01-08

**Authors:** Joana Kästle Silva, Vincent Hervé, Undine S. Mies, Katja Platt, Andreas Brune

**Affiliations:** ^1^ Research Group Insect Gut Microbiology and Symbiosis, Max Planck Institute for Terrestrial Microbiology Marburg Germany

**Keywords:** bifidobacteria, flagellates endosymbiosis, genome reduction, gut microbiota, horizontal gene transfer, termites

## Abstract

Cellulolytic flagellates are essential for the symbiotic digestion of lignocellulose in the gut of lower termites. Most species are associated with host‐specific consortia of bacterial symbionts from various phyla. 16S rRNA‐based diversity studies and taxon‐specific fluorescence in situ hybridization revealed a termite‐specific clade of *Actinomycetales* that colonise the cytoplasm of *Trichonympha* spp. and other gut flagellates, representing the only known case of intracellular *Actinomycetota* in protists. Comparative analysis of eleven metagenome‐assembled genomes from lower termites allowed us to describe them as new genera of *Bifidobacteriaceae*. Like the previously investigated *Candidatus* Ancillula trichonymphae, they ferment sugars via the bifidobacterium shunt but, unlike their free‐living relatives, experienced significant genome erosion. Additionally, they acquired new functions by horizontal gene transfer from other gut bacteria, including the capacity to produce hydrogen. Members of the genus *Ancillula* (average genome size 1.56 ± 0.2 Mbp) retained most pathways for the synthesis of amino acids, including a threonine/serine exporter, providing concrete evidence for the basis of the mutualistic relationship with their host. By contrast, *Opitulatrix* species (1.23 ± 0.1 Mbp) lost most of their biosynthetic capacities, indicating that an originally mutualistic symbiosis is on the decline.

## Graphical abstract

We have characterised several novel genera of *Bifidobacteriaceae* that colonise the cytoplasm of cellulolytic flagellates. Horizontal gene transfer played an important role in their adaptation to the intracellular environment. However, progressive genome erosion led to the loss of biosynthetic capacities that formed the basis of an originally mutualistic symbiosis.

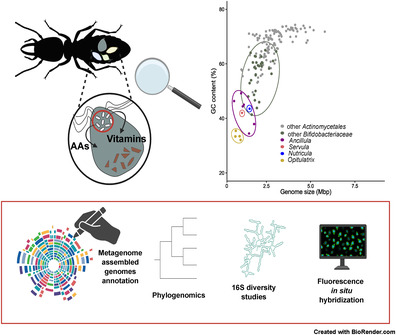

## 1 Introduction

Termites evolved from subsocial cockroaches about 150 million years ago (Chouvenc et al. 2021). With the help of their symbiotic gut microbiota, they digest wood and other lignocellulosic matter (Brune 2014), which lends them a key role in the carbon cycle and other ecosystem services in the tropics and subtropics (Jouquet et al. 2011; Griffiths et al. 2019). Except for the family Termitidae (‘higher termites’), termites from all other families (‘lower termites’) harbour species‐specific assemblages of cellulolytic flagellates that break down wood fibres into compounds that can be assimilated by the host (Brune 2014). The flagellates are themselves colonised by diverse prokaryotic consortia, resulting in a multitiered network of symbiotic interactions (Ohkuma and Brune 2011; Hongoh and Ohkuma 2018).

Symbiotic bacteria are located on the surface of the flagellate cell (ectosymbionts) or colonise the cytoplasm or the nucleus of their flagellate host (endosymbionts) (Brune and Dietrich 2015). These associations are host‐specific and may result in co‐speciation between the partners (Noda et al. 2007; Ikeda‐Ohtsubo and Brune 2009). The endosymbionts were recruited from a wide range of bacterial phyla, including *Bacteroidota* (Noda et al. 2006), *Spirochaetota* (Ohkuma et al. 2015), *Elusimicrobiota* (Stingl et al. 2005; Ohkuma et al. 2007; Ikeda‐Ohtsubo et al. 2007), *Desulfobacterota* (Ikeda‐Ohtsubo et al. 2016), *Verrucomicrobiota* (Sato et al. 2014) and *Bacillota* (Takahashi et al. 2023), but also among *Archaea* (Hongoh and Ohkuma 2018; Kaneko et al. 2024).

The first and so far only case of endosymbiotic *Actinomycetota* in termite gut flagellates is a deep‐branching clade of *Actinomycetales* that colonise certain *Trichonympha* species in dry‐wood termites (Strassert et al. 2012). The draft genome of ‘*Candidatus* Ancillula trichonymphae’, which was assigned a sister position to *Bifidobacteriaceae*, revealed their capacity to ferment xylose, *N*‐acetyl‐glucosamine and glycerol 3‐phosphate through the bifidobacterial pathway (Strassert et al. 2016). However, the diversity of the clade, the presence of related bacteria in other termite lineages, their full metabolic potential and the basis of a potentially mutualistic relationship between the endosymbionts and their flagellate hosts remained unresolved.

To fill this knowledge gap, we investigated the diversity and phylogenetic history of termite‐associated *Bifidobacteriaceae* and the genomic changes involved in their adaptation to an intracellular lifestyle using culture‐independent molecular and bioinformatic techniques. This includes phylogenomic and functional analyses comparing the metagenome‐assembled genomes (MAGs) of termite‐associated *Bifidobacteriaceae* to the genomes of their closest relatives, diversity analysis of 16S rRNA genes from cockroach and termite guts, including capillary‐picked suspensions of termite gut flagellates, and the subcellular localization of the endosymbionts by fluorescence in situ hybridization (FISH). Finally, we classified the new lineages represented by the MAGs as new genera and species under the Code of Nomenclature of Prokaryotes Described from Sequence Data (SeqCode) (Hedlund et al. 2022).

## 2 Results

### 2.1 Phylogeny of Termite‐Associated *Bifidobacteriaceae*

**FIGURE 1:**
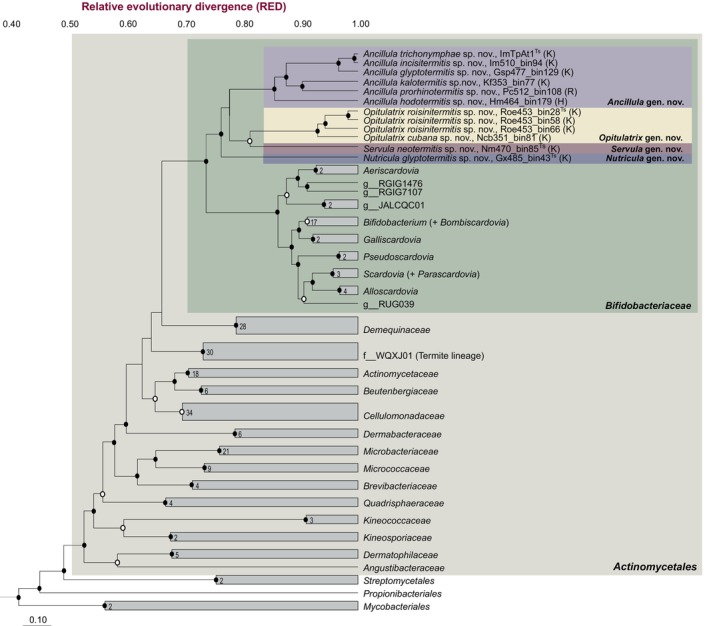
Rank‐normalized phylogeny of *Bifidobacteriaceae*, including type species of all genera validly published under the ICNP. Family‐level lineages of *Actinomycetales* and selected orders of *Actinomycetia* were included for comparison. The maximum‐likelihood tree is based on a concatenated alignment of 120 single‐copy marker genes generated by GTDB‐Tk and was inferred using IQ‐TREE under the LG + F + I + G4 model of evolution. It was normalized using relative evolutionary divergence (RED) values determined with PhyloRank. Bullets on internal nodes indicate ultrafast bootstrap support (●, ≥ 99%; ○, ≥ 95%; 1000 replicates). The number of genomes in the collapsed clades and the type strains of novel genera (^Ts^) are indicated. The family of the host termite is indicated (H, Hodotermitidae; K, Kalotermitidae; R, Rhinotermitidae). For an expanded version of the tree that includes also low‐quality MAGs, see Figure A1.

**FIGURE A1:**
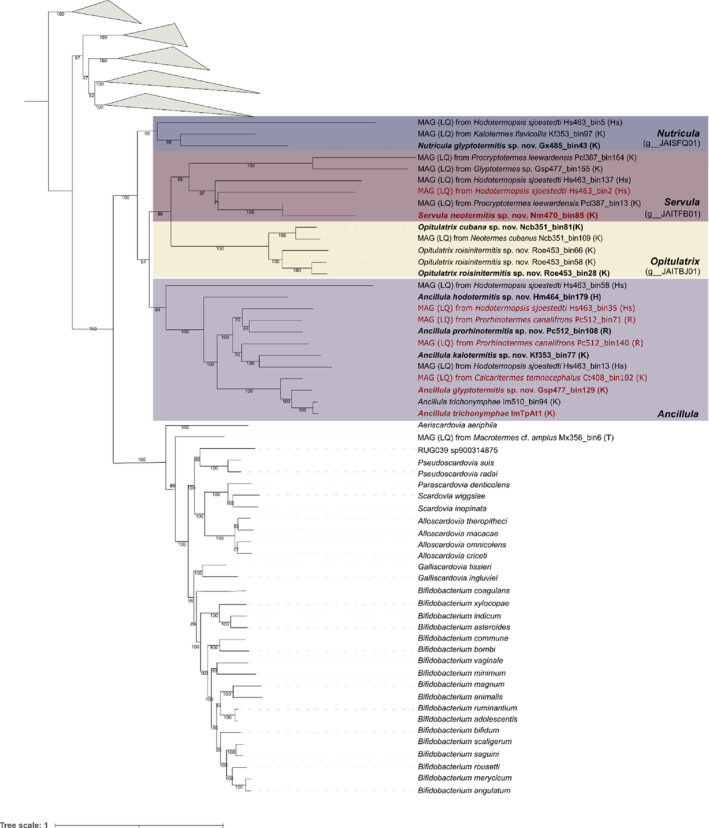
Phylogenomic tree of *Bifidobacteriaceae*, including the names of all genera validly published under the ICNP and low‐quality (LQ) MAGs from termite guts. The maximum‐likelihood tree is based on an alignment of 120 single‐copy marker genes generated by GTDB‐Tk and was inferred with IQ‐TREE under the LG + F + I + G4 model of evolution. Numbers at internal nodes indicate ultrafast bootstrap support (in percent, 1000 replicates). MAGs with 16S rRNA genes are in red typeface, type strains of new species are printed in bold. The host families are indicated in parentheses (A, Archotermopsidae, H, Hodotermitidae; Hs, Hodotermopsidae, K, Kalotermitidae; R, Rhinotermitidae; T, Termitidae).

Among more than 2000 bacterial MAGs from all major termite lineages (Mies et al. 2024), we recovered 8 high‐quality and 3 medium‐quality genomes of termite‐associated *Bifidobacteriaceae*, which were obtained exclusively from lower termites (Table S1). They represent a monophyletic clade in a well‐supported sister position to the members of the family *Bifidobacteriaceae* (Figure 1). The relative evolutionary divergence (RED) values of the individual nodes indicate that the clade comprises four genus‐level lineages that fall—albeit deep‐branching—into the radiation of the family *Bifidobacteriaceae*. One lineage includes ‘*Ca*. Ancillula trichonymphae’, the endosymbiont of *Trichonympha paraspiralis* from *Incisitermes marginipennis* (Strassert et al. 2016), and numerous MAGs from a diverse range of kalotermitid, rhinotermitid and hodotermitid hosts. Each of the remaining lineages is represented by MAGs from only one or two termite species, but their host range increased when low‐quality MAGs were included in the analysis (Figure A1). All lineages are recognised as separate genera also in the most recent version of GTDB (Parks et al. 2022) and were linked to the corresponding clades in the 16S rRNA‐based analysis (see below). Therefore, we propose to classify the members of these lineages in the new genera *Ancillula*, *Opitulatrix* (g__JAITBJ01), *Servula* (g__JAITFB01) and *Nutricula* (g__JAISFQ01) (see Section 3.5).

### 2.2 Genome Characteristics

**FIGURE 2:**
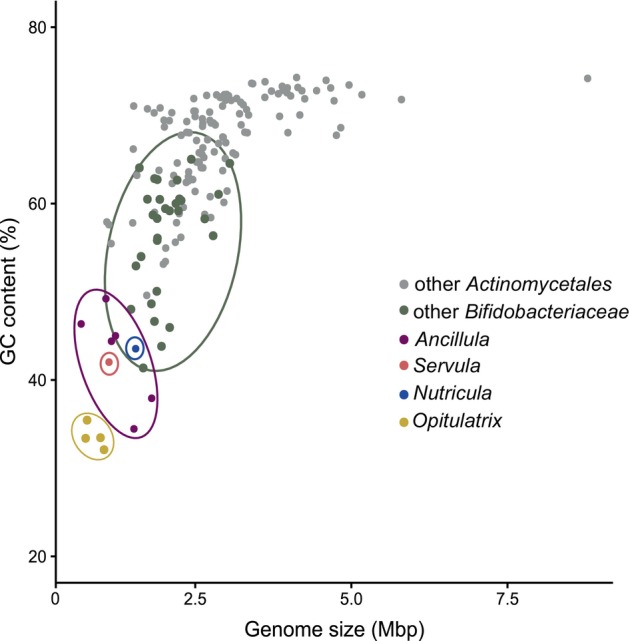
Genome size and genomic GC content of the order *Actinomycetales*, comparing the termite‐associated genera with other members of *Bifidobacteriaceae*.

Each of the new genera is represented by at least one high‐quality genome (Figure 1). The genome sizes of all termite‐associated genera are significantly smaller and their genomic GC content lower than those of other *Bifidobacteriaceae* (Figure 2). After completeness correction with a lineage‐specific marker gene set, the smallest genomes and lowest GC contents (average ± SD) were found in the genus *Opitulatrix* (1.23 ± 0.1 Mbp, 33.6% ± 1.3%), whereas the values for *Ancillula* (1.56 ± 0.2 Mbp, 43.0% ± 5.0%) were in the same range as those for *Servula* (1.61 Mbp, 42.0%) and *Nutricula* (2.01 Mbp, 43.5%). The number of tRNA‐coding genes and anticodons in the respective tRNAs of termite‐associated *Bifidobacteriales* are only slightly lower than in the complete genomes of other *Bifidobacteriaceae* (Table S2), which corroborates the high completeness values of the MAGs. Although the recovery of rRNA genes in the MAGs was poor and their numbers have to be regarded with caution, there is no indication of the presence of more than one rRNA operon in termite‐associated *Bifidobacteriacceae* (Table S2). The number of pseudogenes in the genomes of *Ancillula* (26 ± 19), *Nutricula* (17) and *Servula* (16) is higher than in the genomes of the genus *Opitulatrix* (10 ± 2) (Table S2).

### 2.3 Energy Metabolism

**FIGURE 3:**
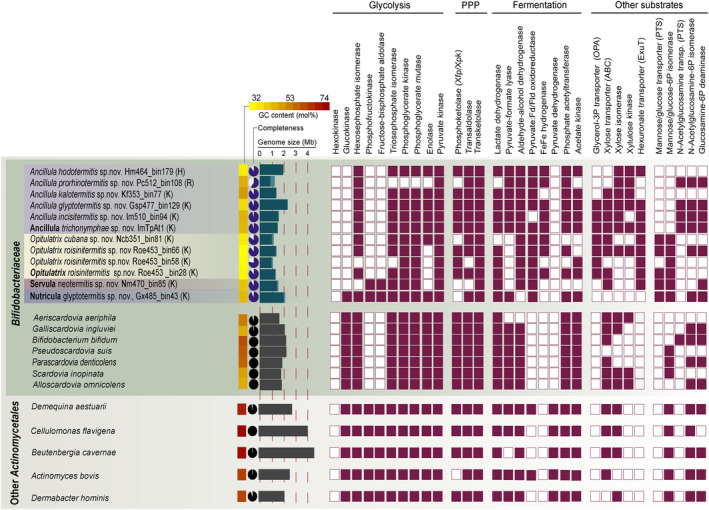
Key enzymes of major catabolic pathways encoded by MAGs from the termite‐associated *Bifidobacteriaceae* and other family members (type species only). Selected representatives of other *Actinomycetales* were included for comparison. Genome size was estimated based on assembly size and completeness of the genomes. More details are shown in (Figures A2 and A3. ABC, ATP‐binding cassette; OPA, organophosphate transporter, PTS, phosphotransferase system; Xfp, xylose‐5‐phosphate/fructose‐6‐phosphate phosphoketolase; Xpk, xylose‐5‐phosphate phosphoketolase.

**FIGURE 4:**
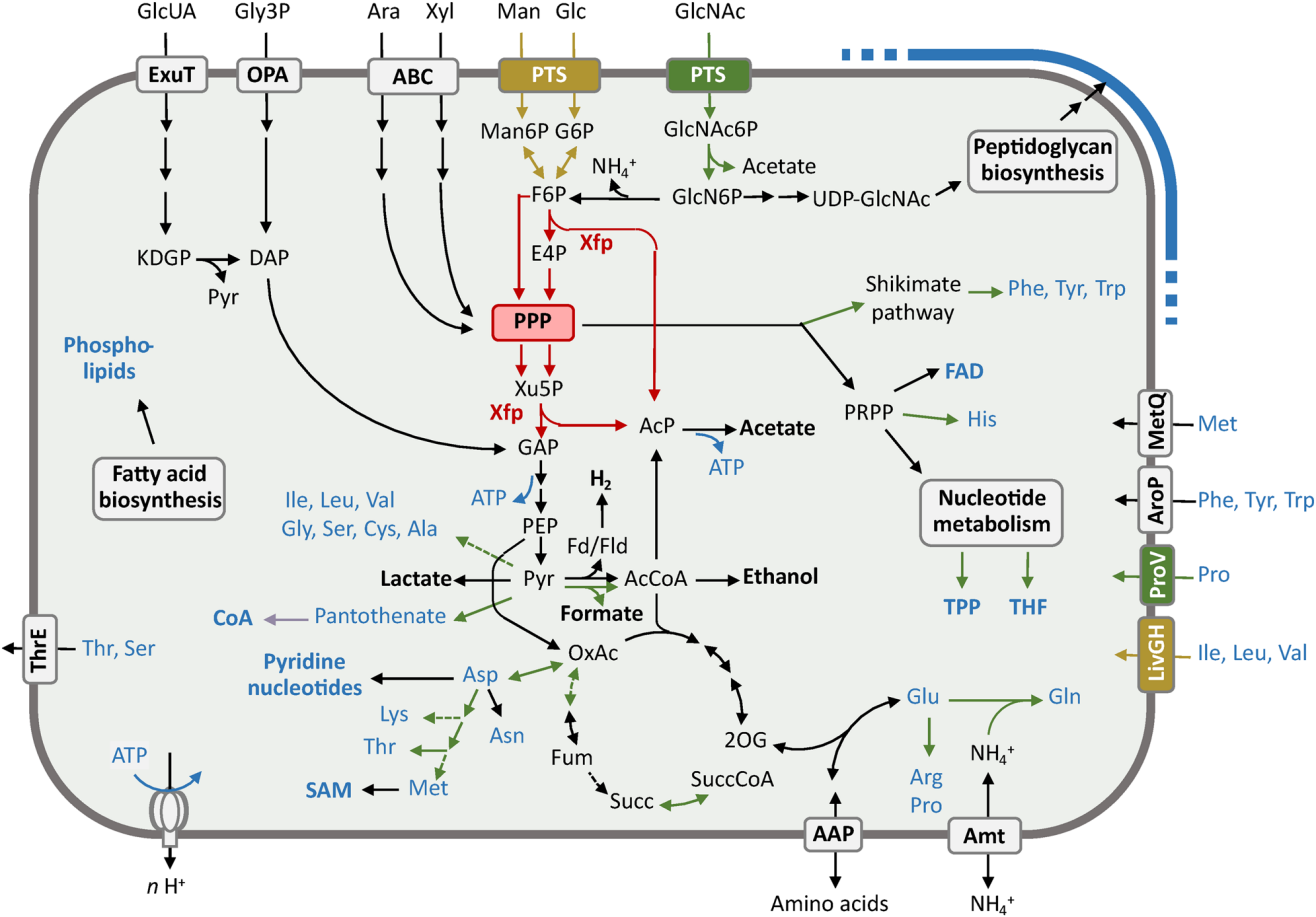
Comparison of the metabolic pathways of the genera *Ancillula* and *Opitulatrix*. Pathways shown in black are present in both genera; the fructose‐6‐phosphate phosphoketolase (F6PPK) pathway (bifidobacteria shunt) is highlighted in red. Elements marked in green are present only in *Ancillula*; those marked in brown are present only in *Opitulatrix*. Dashed arrows indicate that a pathway is not present in all MAGs. Fermentation products are highlighted in bold letters, and products of biosynthetic pathways are shown in blue. Detailed biosynthetic pathways for amino acids and cofactors are shown in Figure A12. Non‐standard abbreviations: AAP, amino acid permease; ABC, ATP‐binding cassette transporter; Amt, ammonium transporter; AroP, aromatic amino acid transporter; Fd/Fld, ferredoxin/flavodoxin; GlcN, glucosamine; GlcNAc, *N*‐acetyl‐glucosamine; GlpT, glycerol‐3‐phosphate transporter; KDPG, 2‐keto‐3‐deoxy‐6‐phosphogluconate; LivGH, branched‐chain amino acid transporter; MetQ, methionine transporter; PLP, pyridoxal 5‐phosphate; PPP, pentose phosphate pathway; PRPP, phosphoribosyl pyrophosphate; PTS, phosphotransferase system; SAM, S‐adenosylmethionine; THF, tetrahydrofolate; ThrE, threonine/serine exporter; Xfp, xylose‐5‐phosphate/fructose‐6‐phosphate phosphoketolase.

**FIGURE A2:**
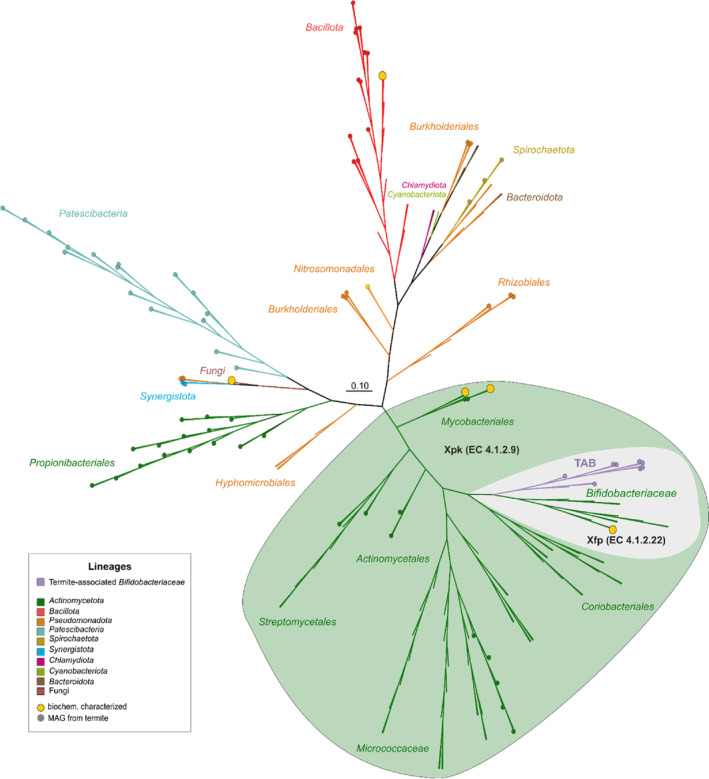
Phylogeny of the bifunctional phosphoketolase (Xfp) of termite‐associated *Bifidobacteriaceae* (TAB) and other family members, and the homologous monofunctional phosphoketolases (Xpk) of *Actinomycetota* and other bacterial phyla. Biochemically characterised homologues (SWISS‐PROT) are highlighted. The maximum‐likelihood tree is based on a curated alignment of 490 amino acid positions and was inferred under the LG + G4 model of evolution. Bootstrap values were omitted for clarity.

**FIGURE A3:**
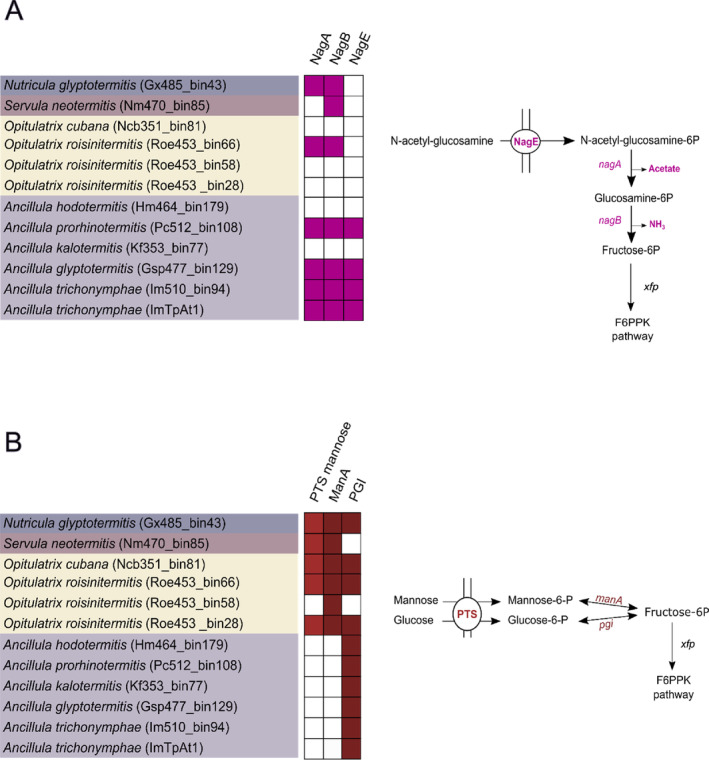
PTS systems for N‐acetyl‐glucosamine (A) and glucose/mannose (B) and their conversion to fructose 6‐phosphate in termite‐associated *Bifidobacteriaceae*.

Members of the genera *Ancillula* and *Opitulatrix* encode all enzymes of the fructose 6‐phosphate phosphoketolase (F6PPK) pathway but lack phosphofructokinase (PFK) and fructose bisphosphate (FBP) aldolase, which are markers of the Embden‐Meyerhoff (EM) pathway (Figure 3). The bifunctional xylulose‐5‐phosphate/fructose‐6‐phosphate phosphoketolase (Xfp), the key enzyme of the F6PPK pathway, clusters with the homologues of other *Bifidobacteriaceae* (Figure A2). *Ancillula* can generate F6P from *N*‐acetyl glucosamine and *Opitulatrix* from glucose and mannose via substrate‐specific uptake and phosphorylation systems (Figures 3, 4 and A3). In addition, both genera encode an ABC transporter for xylose and arabinose and all enzymes for their metabolism via the pentose phosphate pathway (PPP). By contrast, the MAGs of *Nutricula* and *Servula* lack a homologue of Xfp but possess PFK and FBP aldolase, indicating that they metabolise hexoses via the EM pathway (Figure 3).

**FIGURE A4:**
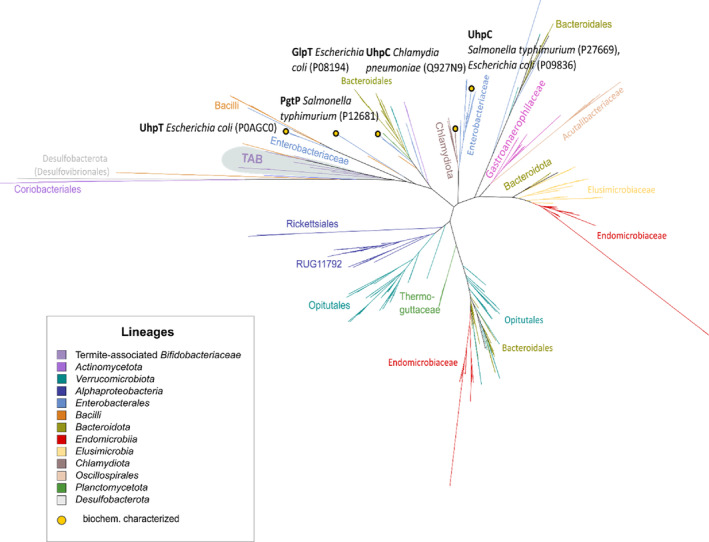
Phylogeny of the organophosphate antiporter (OPA) of termite‐associated *Bifidobacteriaceae* (TAB) and their closest relatives from public reference databases. Biochemically characterised homologues (SWISS‐PROT) are highlighted. The maximum‐likelihood tree is based on a curated alignment of 428 amino acid positions and was inferred under the LG + F + R9 model of evolution. Bootstrap values were omitted for clarity.

Members of the genera *Ancillula* and *Opitulatrix* also encode a glycerol 3‐phosphate transporter (GlpT) of the organophosphate: inorganic phosphate antiporters (OPA) family, which allows the uptake of sugar phosphates from the host cell. The homologues from this family include only a few biochemically characterised enzymes, such as the phosphoglycerate transporter (PgpT) of *Salmonella typhimurium* and the hexose 6‐phosphate transporters of *Escherichia coli* (UhpT) and *Chlamydia pneumoniae* (UhpC) (Figure A4); therefore, their exact substrate specificity remains uncertain. Except for *Nutricula*, termite‐associated *Bifidobacteriaceae* also encode the enzymes required for the uptake and metabolism of hexuronic acids via the 2‐keto‐3‐deoxy‐phosphogluconate (KDPG) pathway (Figures 3 and 4).

**FIGURE A5:**
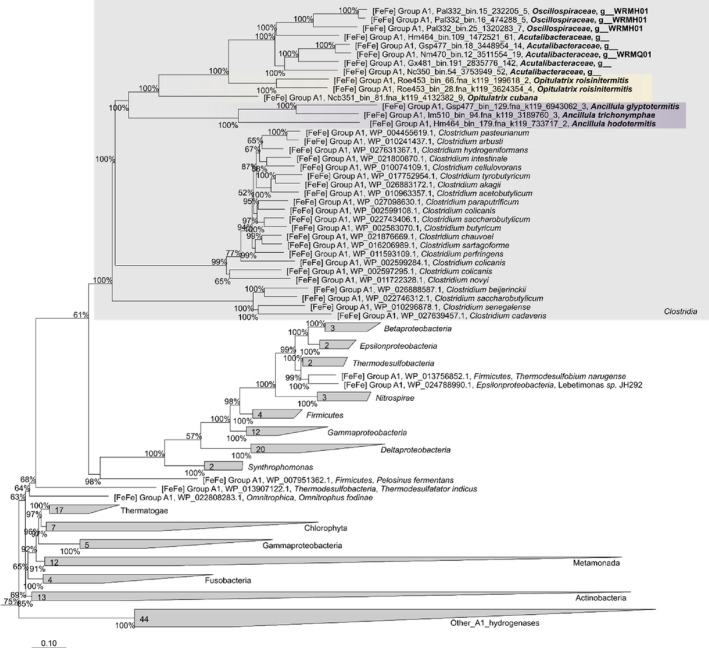
Phylogeny of Group A [FeFe] hydrogenases in the genus *Ancillula* and *Opitulatrix* and their homologues in public databases. Homologues from MAGs of termite gut microbiota are marked in bold. The maximum‐likelihood tree is based on a curated alignment of 281 amino acid positions and was inferred under the LG + R8 model of evolution. Numbers at internal nodes indicate ultrafast bootstrap support (1000 replicates).

**FIGURE A6:**
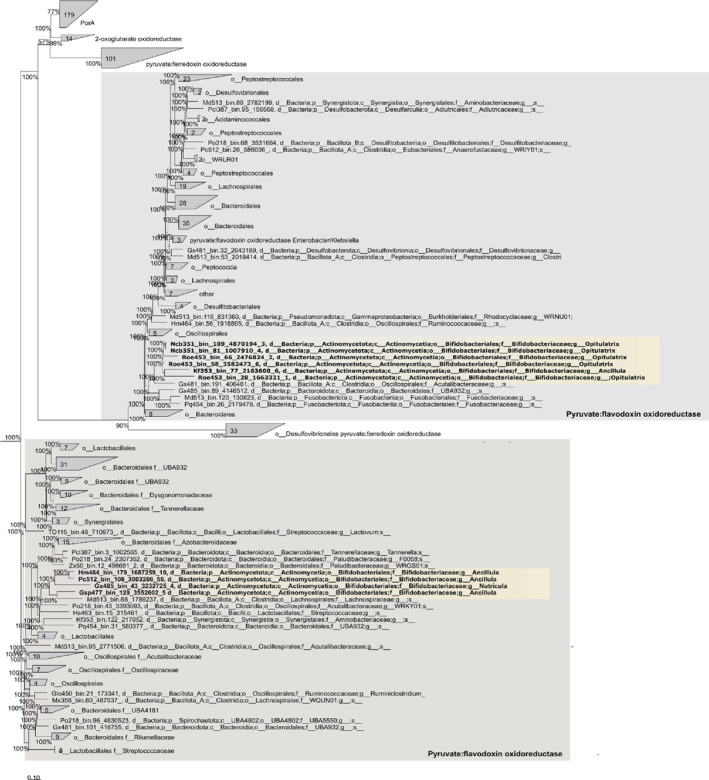
Phylogeny of PFOR in termite‐associated *Bifidobacteriaceae* and their homologues from public databases. The maximum‐likelihood tree is based on a curated alignment of 323 amino acid positions and was inferred under the LG + R5 model of evolution. Numbers at internal nodes indicate ultrafast bootstrap support (1000 replicates).

All termite‐associated *Bifidobacteriaceae* encode lactate dehydrogenase and a pyruvate–ferredoxin/flavodoxin oxidoreductase (PFOR), indicating that pyruvate can be reduced to lactate or oxidised to acetyl‐CoA. In addition, members of *Ancillula* and *Nutricula* may produce acetyl‐CoA via pyruvate–formate lyase. Almost all MAGs encode a bifunctional alcohol/acetaldehyde dehydrogenase (AdhE) that reduces acetyl‐CoA to ethanol. This suggests that the main fermentation products are the same as those of other *Bifidobacteriaceae*, which include acetate, lactate, formate, and ethanol (de Vries and Stouthamer 1968; Palframan, Gibson, and Rastall 2003). The presence of PFOR and a [FeFe] hydrogenase of subgroup A1, which are absent from all other *Bifidobacteriaceae*, in members of *Ancillula* and *Opitulatrix* indicate the capacity to form H_2_ as an additional fermentation product (Figure 4). The phylogenies of the [FeFe] hydrogenase (Figure A5) and PFOR (Figure A6) suggest a clostridial origin. The PFOR sequences of *Ancillula* and *Opitulatrix* are most closely related to flavodoxin‐dependent homologues, making it likely that they use flavodoxin rather than ferredoxin as a low‐potential electron carrier.

Like all *Bifidobacteriaceae*, the termite‐associated genera lack pyruvate dehydrogenase and have an incomplete TCA cycle (Figures 3 and 4). The general absence of biosynthetic pathways for cytochromes and quinones from all MAGs is consistent with the anaerobic metabolism characteristic for *Bifidobacteriaceae*. If ATP is generated exclusively via substrate‐level phosphorylation, the F‐type ATPase of *Bifidobacteriaceae* is not required for ATP synthesis but rather for the maintenance of the electrochemical membrane potential, as in other bacteria with an obligately fermentative metabolism (Buckel and Thauer 2013).

### 2.4 Anabolic Pathways

**FIGURE 5:**
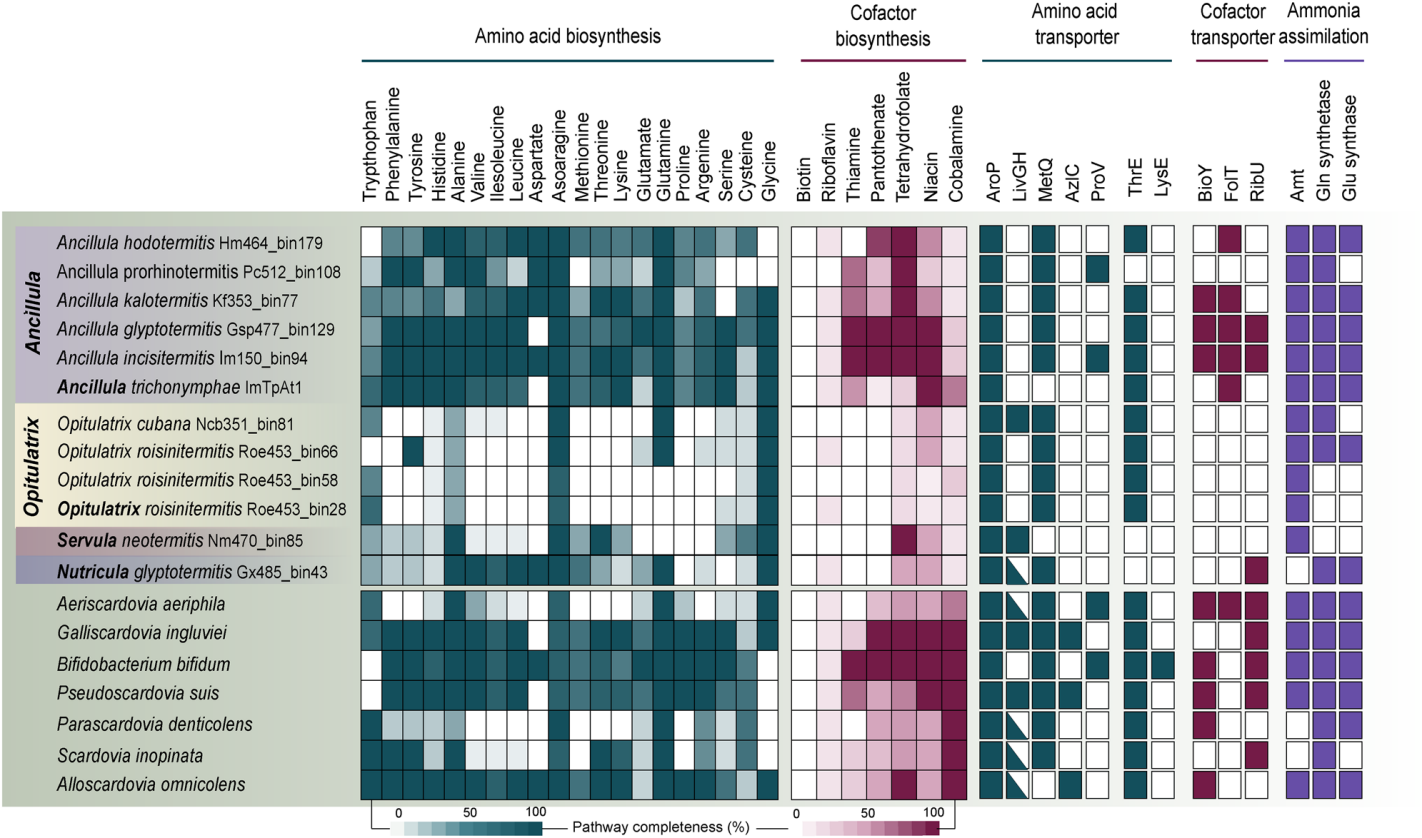
Capacities for biosynthesis and transport of amino acids and co‐factors, and key genes for the assimilation of ammonia of termite‐associated *Bifidobacteriaceae* and other family members (type species only). The full pathways and other details are shown in (Figures A7 and A12). Amt, ammonium transporter; AroP, aromatic amino acid transporter; AzlC, branched‐chain amino acid transporter; BioY, biotin transporter; FolT, folate transporter; LysE, lysine exporter; LivGH, branched‐chain amino acid transporter; MetQ, methionine transporter; ProV, glycine/proline transporter; RibU, riboflavin transporter; ThrE, threonine/serine exporter.

**FIGURE A7:**
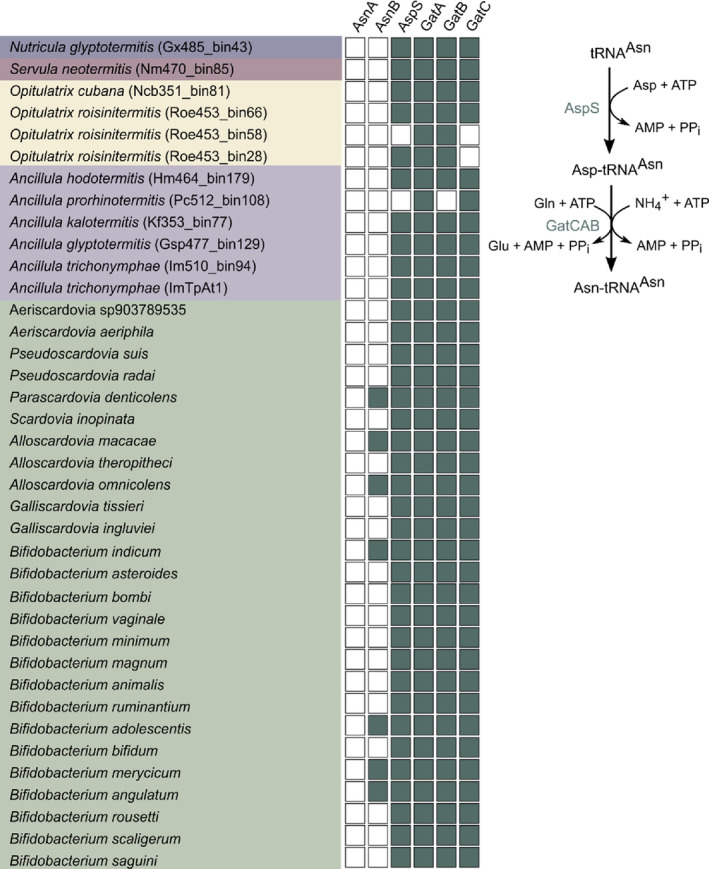
Enzymes of asparagine biosynthesis encoded by termite‐associated *Bifidobacteriaceae* and other family members. Abbreviations: AsnA, aspartate–ammonia ligase; AsnB, asparagine synthetase B; AspS, aspartate–tRNA ligase; GatABC, aspartyl/glutamyl‐tRNA(Asn/Gln) amidotransferase.

While members of the genera *Ancillula* and *Nutricula* have retained the capacity for *de novo* biosynthesis of most proteinogenic amino acids, the corresponding pathways are mostly incomplete or absent among members of the genera *Opitulatrix* and *Servula* (Figure 5). Most termite‐associated genera encode ABC transporters for methionine (MetQ) and aromatic amino acids (AroP) and an amino acid permease (AAP) with a broad substrate range. Although all MAGs of *Opitulatrix* and *Servula* lack the biosynthetic potential for branched‐chain amino acids, the corresponding ABC transporter (LivGH) is encoded only by *Opitulatrix cubana* and *Servula enteritis*. Like other *Bifidobacteriaceae*, all genera lack asparagine synthetase (AsnAB) but (except for *Opitulatrix*) encode an unusual aspartyl‐tRNA synthetase (AspS2) that loads an aspartyl residue onto tRNA^Asn^, where it is subsequently converted to an asparaginyl residue by aspartyl‐tRNA amidotransferase (GatCAB) (Figure A7) (Min et al. 2002).

**FIGURE A8:**
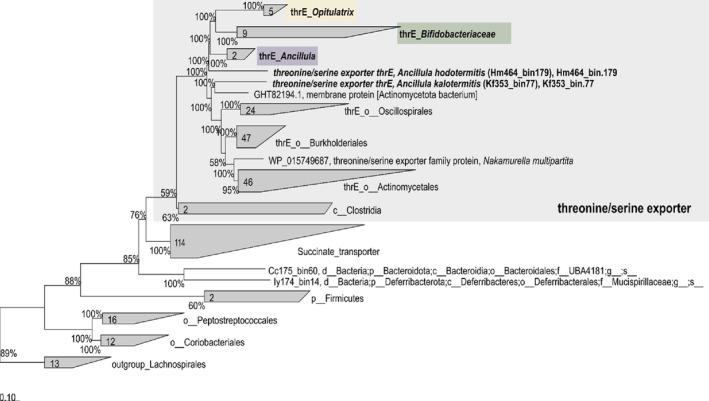
Phylogeny of the threonine/serine exporter (ThrE) of termite‐associated *Bifidobacteriaceae* and its homologues in public databases. The maximum‐likelihood tree is based on a curated alignment of 259 amino acid positions and was inferred under the LG + F + R6 model of evolution. Numbers at internal nodes indicate ultrafast bootstrap support (1000 replicates).

Almost all MAGs of *Ancillula* and *Opitulatrix* encode a threonine/serine exporter (ThrE). It is most closely related to homologues from other *Bifidobacteriaceae* (Figure A8), indicating that the gene was present already in the common ancestor of the family but lost in *Servula* and *Nutricula*. Many MAGs encode an ammonium transporter (Amt), glutamine synthetase and a putative transaminase, indicating the capacity to assimilate ammonia, whereas others must acquire combined nitrogen in the form of amino acids (Figure 4). Genes encoding nitrogenase (NifHDK) are absent from all MAGs.

The termite‐associated genera cannot generally synthesise biotin, riboflavin, and (unlike other *Bifidobacteriaceae*) also cobalamin (Figure 5). *Ancillula glyptotermitis* and *A. incisitermitis* are exceptions, retaining the potential for the *de novo* synthesis of most B vitamins (e.g., niacin, tetrahydrofolate, pantothenate, and thiamine). However, transport systems for biotin (BioY), riboflavin (RibU) and folate (FolT) are absent from all genera but *Ancillula*.

**FIGURE A9:**
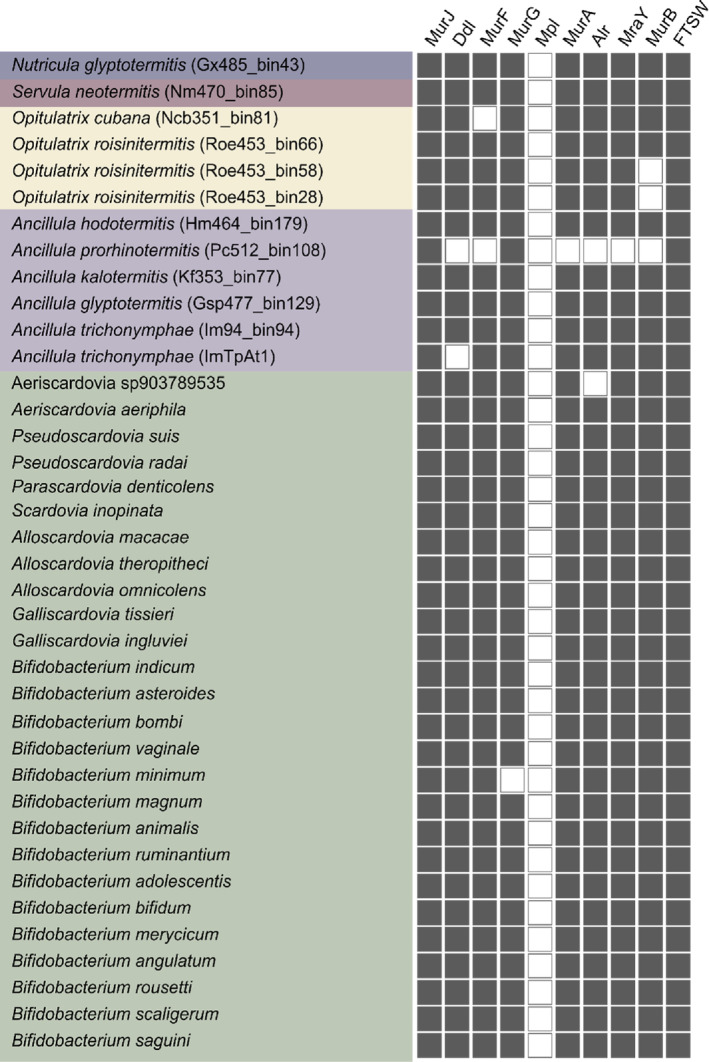
Enzymes of murein biosynthesis encoded by termite‐associated *Bifidobacteriaceae* and other family members. Abbreviations: MurJ, Lipid II flippase; Ddl, D‐alanine–D‐alanine ligase; MurF, UDP‐N‐acetylmuramoyl‐tripeptide–D‐alanyl‐D‐alanine ligase; Mpl, UDP‐N‐acetylmuramate–L‐alanyl‐gamma‐D‐glutamyl‐meso‐2,6‐diaminoheptandioate ligase; MurA, UDP‐N‐acetylglucosamine 1‐carboxyvinyltransferase; Alr, Alanine racemase; MraY, phospho‐N‐acetylmuramoyl pentapeptide transferase; MurB, UDP‐N‐acetylenolpyruvoylglucosamine reductase; FTSW, peptidoglycan glycosyltransferase.

Peptidoglycan biosynthesis is conserved in all lineages of termite‐associated *Bifidobacteriaceae* (Figure A9). An exception is the pathway for diaminopimelic acid (l‐DAP) production, which is conserved only in the genus *Ancillula*, indicating a requirement for lysine in all other lineages. The capacity to synthesise murein is in agreement with the thick cell wall observed in electron micrographs of *Ancillula trichonymphae* (Strassert et al. 2012).

### 2.5 Other Genomic Features

**FIGURE A10:**
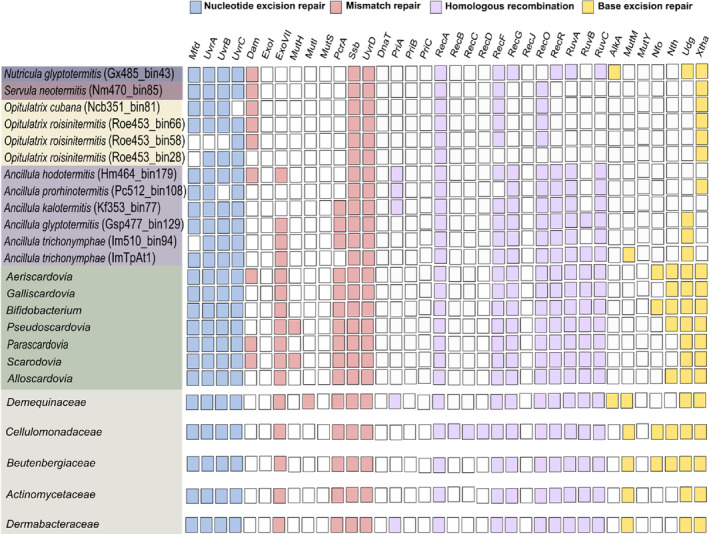
Elements of different DNA repair mechanisms encoded by *Bifidobacteriacea*e and other *Actinomycetales*.

The annotation of the genes involved in DNA repair mechanisms revealed that termite‐associated *Bifidobacteriaceae* lack many of the genes that in *Escherichia coli* are involved in fixing DNA damage, particularly in mismatch repair (MMR) pathways (Figure A10). However, numerous gene loci involved in mismatch repair (*mutS*, *mutL*) and homologous recombination (*recABC*, *recD*, *recF*, *recG*, *recJ*, *recO*, *recR*) are generally absent from *Bifidobacteriaceae* and other *Actinomycetales*. Transposable elements, prophages, and CRISPR structures and their associated *cas* genes were not detected in any of the MAGs.

A search for gene clusters involved in secondary metabolite biosynthesis using automated annotation pipelines was negative. We found no evidence for the biosynthesis of bacteriocins such as bifidins or lantibiotics that are produced by other *Bifidobacteriaceae* (Cheikhyoussef et al. 2010; Lee, Li, and O'Sullivan 2011). Extracellular structures like sortase‐dependent pili and tight adherence pili, which are essential for host colonisation in the genus *Bifidobacterium* (Alessandri, van Sinderen, and Ventura 2021), are not encoded by the termite‐associated genera. Likewise, there was no indication of a type IV secretion system, which seems to be present in other bifidobacterial species (Guglielmetti et al. 2014).

The catalogue of carbohydrate‐active enzymes (CAZymes) encoded by termite‐associated *Bifidobacteriaceae* reveals the presence of several glycosyl hydrolases (GHs) and glycosyl transferases (Table S3). The majority of the GHs belong to the families GH13, GH1 and GH3 and are involved in glycogen metabolism. Only very few of the gene products were predicted to encode signal peptides that would indicate secretion into the extracellular space. GHs acting on polymers of plant cell walls were not detected.

### 2.6 Diversity and Potential Flagellate Hosts

**FIGURE 6:**
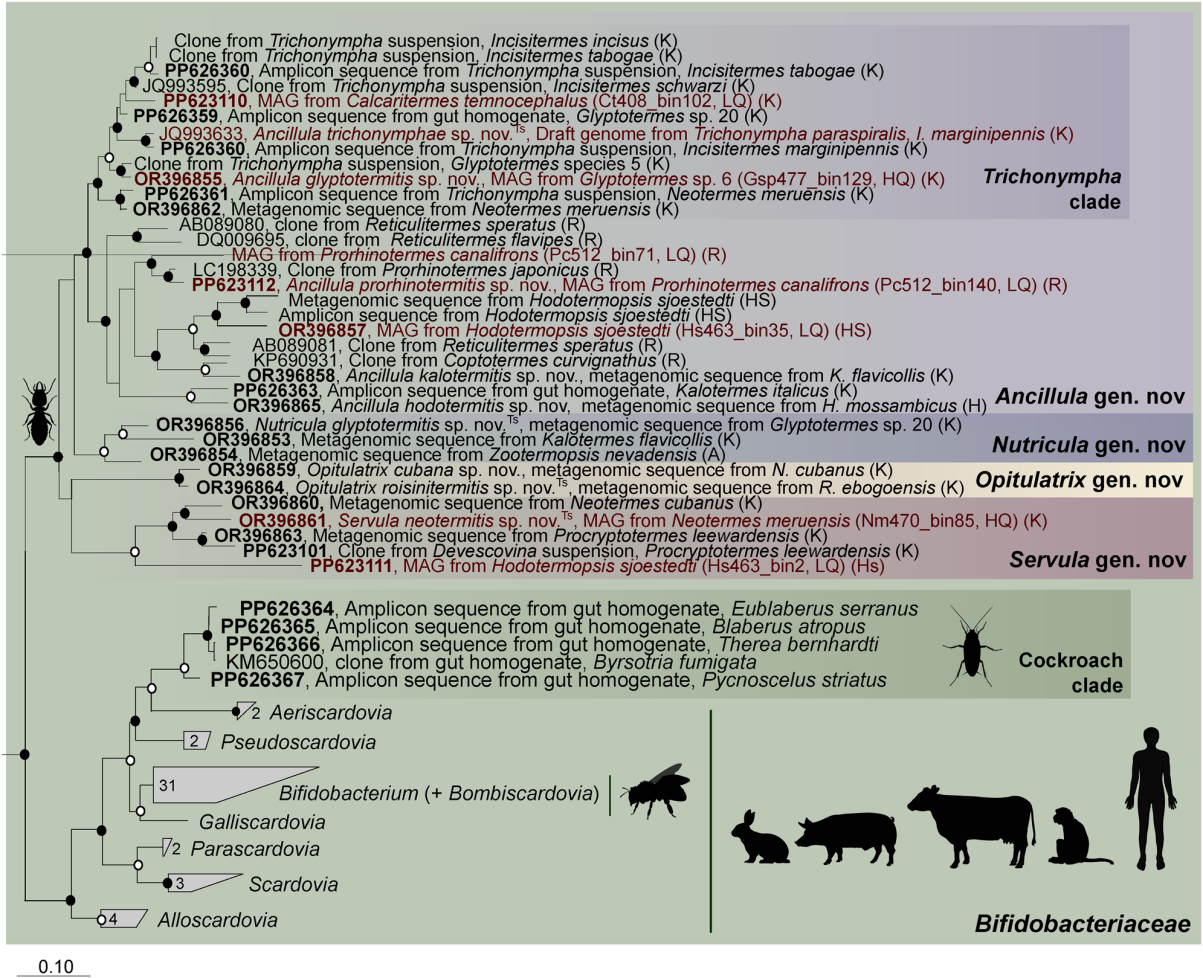
16S rRNA‐based phylogeny of *Bifidobacteriaceae*, illustrating the relationship of the phylotypes recovered from whole‐gut libraries of termites and cockroaches and capillary‐picked suspensions of termite gut flagellates to the type species of all other genera. Accession numbers of novel sequences are shown in bold. The number of sequences in the collapsed clades and the type strains of novel genera (Ts) are indicated. The host species are indicated (termite family in parentheses: A, Archotermopsidae, H, Hodotermitidae; Hs, Hodotermopsidae, K, Kalotermitidae; R, Rhinotermitidae). Sequences from MAGs are marked in red. The maximum‐likelihood tree is based on a curated alignment of near full‐length 16S rRNA genes (1462 positions) and was generated using IQ‐TREE under the SYM + R4 model of evolution. Bullets on internal nodes indicate ultrafast bootstrap support (●, ≥ 99%, ○, ≥ 95%, 1000 replicates). Other *Actinomycetales* were used as outgroups. Symbols created with BioRender.com.

To assess the diversity of termite‐associated *Bifidobacteriaceae* and their potential association with specific flagellates, we compiled previously unpublished 16S rRNA gene sequences from clone libraries and long‐read amplicon libraries of termite and cockroach gut microbiota obtained in our laboratory, including those from numerous capillary‐picked suspensions of termite gut flagellates (Table S4). Together with 16S rRNA genes extracted from metagenomic libraries and MAGs from a wide range of termites (Tables S1 and S2) (Hervé et al. 2020; Mies et al. 2024), the dataset considerably expanded the known diversity of the termite‐associated *Bifidobacteriaceae* (Figure 6).

Phylogenetic analysis revealed that all sequences of *Bifidobacteriaceae* from termites formed a monophyletic group (Figure 6). The largest clade comprised sequences of the genus *Ancillula*, represented by the 16S rRNA genes obtained from the MAGs that were assigned to this genus (Figures 1 and A1) and related phylotypes from clone or amplicon libraries and metagenomic dataset of other termites. One subclade consists exclusively of phylotypes from flagellate suspensions of *Trichonympha* spp. from dry‐wood termites (Kalotermitidae) or whole‐gut samples of Kalotermitidae that harbour flagellates of this genus. They include *Ancillula trichonymphae*, the endosymbiont of *Trichonympha* spp. from *Incisitermes marginipennis* and *Incisitermes schwarzi* (Strassert et al. 2012), and novel phylotypes from *Trichonympha* suspensions of *I. tabogae* and *I. incisus* . The reads assigned to the genus *Ancillula* dominate the amplicon libraries of capillary‐picked *Trichonympha* suspensions from the genus *Incisitermes* (16%–81% of the bacterial reads) but are less abundant in *Trichonympha* suspensions from *Glyptotermes* sp. 5 (0.38%) (Table S5). The other subclades of *Ancillula* consist mostly of phylotypes from whole‐gut samples of other termite species, including *Kalotermes italicus* and *Neotermes cubanus*. Here, a larger proportion of *Ancillula* reads (0.99%–3.10%) in whole‐gut libraries of *K. italicus* than in the associated suspensions of *Joenia* spp. (0.18%–0.68%) suggest a contamination with bacterial symbionts of other gut flagellates, so that the identity of their host remains elusive.

The other clades comprised sequences that were assigned to the genera *Opitulatrix*, *Servula* and *Nutricula*, based on the 16S rRNA genes obtained from the corresponding MAGs (Figures 1 and A1) and related phylotypes from amplicon libraries and metagenomic datasets of other termites. In all cases, the termite‐specific *Bifidobacteriaceae* account only for a small number of reads in the respective libraries (Table S5). None of these libraries was derived from capillary‐picked *Trichonympha* suspensions or whole‐gut samples of termites harbouring these flagellates, indicating that they colonise flagellates of other genera. The genus *Servula* comprises a phylotype from a flagellate suspension of *Devescovina* (Cristamonadida) species from *Procryptotermes leewardensis* (0.04%), and the genus *Nutricula* was associated, albeit at very low abundance (0.14%), with another cristamonadid flagellate, *Joenia annectens*, from *Kalotermes italicus* (Table S5). Sequences of the genus *Opitulatrix* were linked to the phylogenomic dataset by MAGs from *Neotermes cubanus* and *Roisinitermes ebogensis* (0.06% in a whole‐gut sample of *Neotermes cubanus*).

In addition, we recovered a novel genus‐level lineage of uncultured *Bifidobacteriaceae* from whole‐gut libraries of several cockroaches (Table S5). The phylotypes did not cluster with the termite‐associated lineages but fell into the radiation of other *Bifidobacteriaceae*, with members of the genus *Aeriscardovia* as the closest relatives (Figure 6). They were represented in all cockroach samples investigated but made up only a small fraction (0.01%–1.1%) of the reads in the respective libraries (Table S5).

### 2.7 Subcellular Localization

**FIGURE 7:**
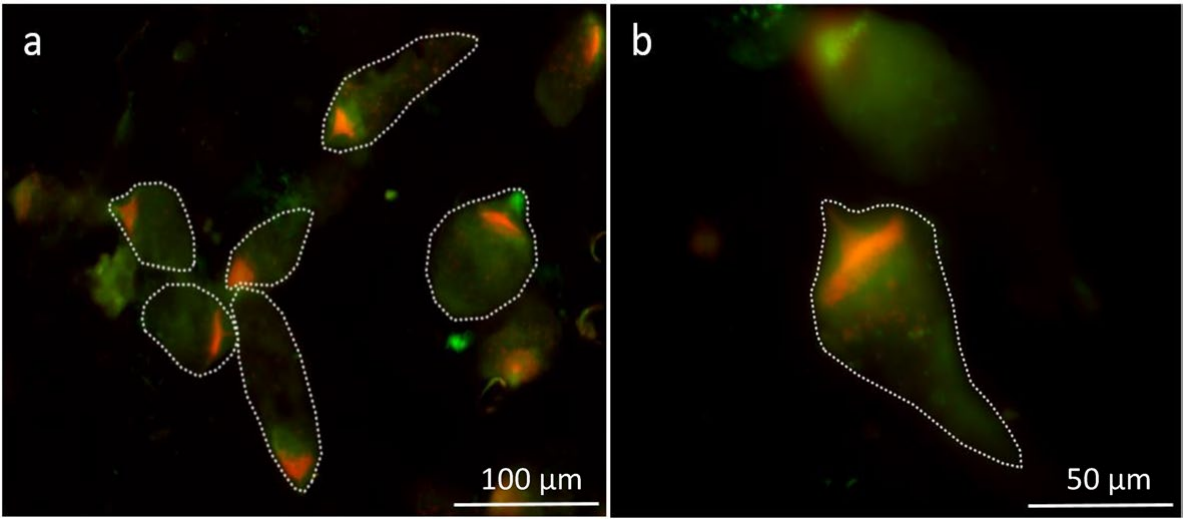
Fluorescence in situ hybridization analysis of the hindgut content of *Incisitermes tabogae* (A) and *I. incisus* (B). Bacterial cells were hybridised with the general *Bacteria* probe EUB338 (Fam‐labelled, green) and the probe ACT490 specific for the termite‐associated lineages (Cy3‐labelled, orange). Outlines of *Trichonympha* cells were drawn based on phase‐contrast images of the same slides. Scale bars: 100 μm (A) and 50 μm (B).

The intracellular location of *Ancillula trichonymphae* in the region is anterior to the nucleus in *Trichonympha* spp. of *Incisitermes schwarzi* and *I. marginipennis* has been documented already with FISH (Strassert et al. 2012). Using the same protocol, we assessed the subcellular localization patterns of other termite‐associated *Bifidobacteriaceae* in fixed hindgut content of several kalotermitids using dual hybridization with general bacterial (Eub‐338, fam‐labelled) and taxon‐specific (Act‐490, cy3‐labelled) probes. In the case of *Incisitermes incisus* and *Incisitermes tabogae*, we observed a large number of bacterial cells hybridising with the oligonucleotide probe Act‐490 in *Trichonympha* spp. associated with the respective termite hosts (Figure 7), showing the same accumulation at the anterior pole of the host cell as previously documented for other *Incisitermes* spp. (Strassert et al. 2012). Simultaneous hybridization with a general bacterial probe (EUB338) indicated colonisation of the *Trichonympha* cells also by other bacterial symbionts. Although the target region of probe Act‐490 is conserved among all representatives of termite‐associated *Bifidobacteriaceae*, the fixed hindgut content of *Glyptotermes* species 5, *Kalotermes flavicollis* and *Kalotermes italicus* showed only cells hybridising with the bacterial probe.

## 3 Discussion

Our study documents the diversity of termite‐associated *Bifidobacteriaceae*, their distribution across a wide range of termite species, and their association with specific gut flagellates. Comparative genome analysis demonstrated the presence of four genus‐level lineages that share many metabolic traits with *Bifidobacteriaceae* associated with mammalian hosts. However, their intracellular lifestyle led to strong genome erosion and the acquisition of new functions from other termite gut microbiota via horizontal gene transfer (HGT).

### 3.1 Catabolism by Termite‐Associated *Bifidobacteriaceae*

Members of the family *Bifidobacteriaceae* are distinguished from other *Actinomycetales* by the lack of pyruvate dehydrogenase, an incomplete TCA cycle, and the absence of a respiratory metabolism (Genilloud 2017). The termite‐associated genera are no exception. Like other *Bifidobacteriaceae*, the members of the genera *Ancillula* and *Opitulatrix* metabolise hexoses via the fructose 6‐phosphate phosphoketolase (F6PPK) pathway (Scardovi 1965). This pathway, also known as ‘bifidobacterium shunt’ generates 0.5 ATP more per glucose than the Embden–Meyerhof (EM) pathway (Schramm, Klybas, and Racker 1958). The bifunctional phosphoketolase (Xfp) of *Bifidobacteriaceae*, which uses both xylulose 5‐phosphate and fructose 6‐phosphate as substrates (Grill, Crociani, and Ballongue 1995), forms a monophyletic group in the radiation of the monofunctional xylulose‐5‐phosphate phosphoketolase (Xpk) of *Actinomycetota* (Figure A2), which serves exclusively in the metabolism of pentoses (Meile et al. 2001; Xiong et al. 2015). The genera *Servula* and *Nutricula* are notable exceptions. They lack Xfp but possess phosphofructokinase and fructose biphosphate aldolase (Figure 3), suggesting that they retained the ancestral trait of *Actinomycetales* and metabolise hexoses via the EM pathway (Genilloud 2017).

While members of the termite‐associated clade probably form the same fermentation products as other *Bifidobacteriaceae*, they differ in their capacity to produce hydrogen. In addition to pyruvate–formate lyase, which is present in most *Bifidobacteriaceae*, the termite‐associated genera encode PFOR and an [FeFe] hydrogenase of subgroup A1 (Figure 3), which are probably coupled by flavodoxin and allow the generation of additional ATP by substrate‐level phosphorylation, provided that the hydrogen partial pressure of the environment is low enough to release reducing equivalents as H_2_ (Schuchmann, Chowdhury, and Müller 2018). Measurements of the intestinal H_2_ partial pressure showed that *Kalotermes flavicollis* and *Incisitermes marginipennis* accumulate H_2_ in the hindgut paunch colonised by flagellates, while *Neotermes* and other kalotermitids do not (Desai and Brune 2012).

Many host‐associated *Bifidobacteriaceae* that colonise the gastrointestinal tract of mammals produce CAZymes that are secreted into the extracellular space and involved in the breakdown of complex polysaccharides (Pokusaeva, Fitzgerald, and van Sinderen 2011). However, the majority of GHs encoded by termite‐associated *Bifidobacteriaceae* are α‐glucosidases of the family GH13 (Table S3), which are common also in bifidobacterial species colonising the human gut (Milani et al. 2015) and involved in the breakdown of amylose and glycogen. We found no evidence for extracellular xylanases, arabinofuranosidases or xylosidases that break down plant‐derived glycans in other bifidobacterial species (Kelly, Munoz‐Munoz, and van Sinderen 2021; Orihara et al. 2023). This is in agreement with the hypothesis that endosymbiotic *Bifidobacteriaceae* use monosaccharides, hexuronates, and sugar phosphates as energy substrates.

### 3.2 Genome Erosion

Termite‐associated *Bifidobacteriaceae* have significantly smaller genomes and a lower genomic GC content than their gut‐associated but free‐living relatives. While the copy number of rRNA‐specifying operons in the genomes of other *Bifidobacteriaceae* ranges between two and five (Bottacini et al. 2014), the termite‐associated lineages seem to possess only one set of rRNA genes. Although rRNA genes are often excluded during the binning procedure, a low copy number is part of the genome streamlining common in intracellular bacteria and would be in agreement also with the low growth rates of intracellular symbionts. Genome erosion is most pronounced in the genus *Opitulatrix*, whose genome sizes and GC content are substantially lower than those of their free‐living relatives (Figure 2), as observed also for other endosymbionts of termite gut flagellates (Mies et al. 2024). Intracellular symbionts are prone to harmful genetic drift, where increased mutation rates and the relaxed selection of non‐essential traits lead to progressive gene loss (McCutcheon and Moran 2012). It is generally assumed that the primary cause of genome erosion is the loss of DNA repair mechanisms and the subsequent accumulation and fixation of point mutations (Hershberg and Petrov 2010). In intracellular *Mycoplasma* and *Spiroplasma* species, the decreased gene repertoire in the mismatch repair pathways has been linked to increased substitution rates and rapid genome evolution (Delaney et al. 2012; Gerth et al. 2021). However, the general absence of many genes involved in DNA repair also from other *Actinomycetales* (Figure A10) indicates that these are not gene losses related to an intracellular lifestyle. Rather, the presence of a non‐canonical mismatch repair pathway in *Actinobacteria* and *Archaea* (Castañeda‐García et al. 2017) indicates that actinobacteria might have further DNA repair mechanisms that remain elusive.

Despite the considerable genome reduction, the genera *Ancillula* and *Nutricula* have retained many biosynthetic pathways present also in host‐associated but free‐living *Bifidobacteriaceae* (Figures 3 and 5). By contrast, members of the genera *Opitulatrix* and *Servula* have lost the capacity for the *de novo* biosynthesis of most proteinogenic amino acids, which leaves them fully reliant on the acquisition of amino acids from the host environment (Figure 5).

### 3.3 Acquisition of New Functions

**FIGURE A11:**
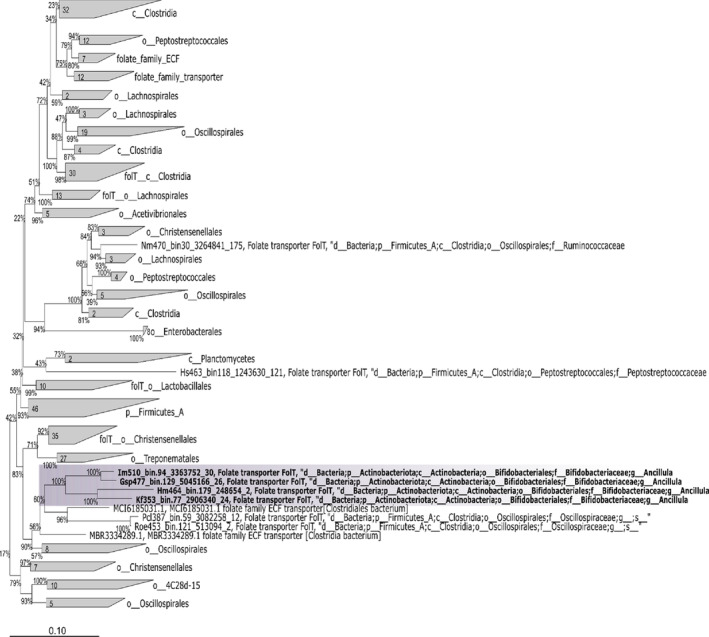
Phylogeny of the folate transporter (FolT) encoded by members of the genus *Ancillula* and its homologues from public databases. The maximum‐likelihood tree is based on a curated alignment of 324 amino acid positions and was inferred under the LG + G4 model of evolution. Numbers at internal nodes indicate ultrafast bootstrap support (1000 replicates).

Termite‐associated *Bifidobacteriaceae* possess several functions that are most likely acquired by horizontal gene transfer from other gut bacteria. One example is PFOR, which is generally absent from *Actinomycetales*. The homologues encoded by the termite‐associated clade fall into the radiation of pyruvate–flavodoxin oxidoreductases from two groups of uncultured *Lachnospiraceae* and *Acutalibacteraceae* (*Clostridia*) that comprise many representatives from termite guts, including other endosymbionts of flagellates, such as members of the genus *Trichonympha* (Figure A6) (Takahashi et al. 2023; Mies et al. 2024). This is in accordance with the phylogeny of the accompanying subgroup A1 [FeFe] hydrogenase, in which *Acutalibacteraceae* homologues from the termite gut also share the phylogenetic neighbourhood with sequences from *Ancillula* and *Opitulatrix* (Figure A5). In that context, it is important that also the folate transporter (FolT) found in the genus *Ancillula* has been acquired by lateral gene transfer from other gut microbiota. Notably, phylogenetic analysis places it among homologues in the family *Oscillospirales*, with *Acutalibacteraceae* again as close relatives (Figure A11).

Another example is the sugar‐phosphate transporter (OPA) present in *Ancillula* and *Opitulatrix*, which is phylogenetically embedded among homologues from MAGs of *Lactobacillaceae* that were also obtained from termite guts (Figure A4). Although its exact substrate specificity is uncertain, any OPA would provide the capacity to utilise sugar phosphates, which are in ample supply in the cytoplasm of the host. OPAs are considered a hallmark of an intracellular lifestyle (Jeckelmann and Erni 2020). Their independent acquisition also by members of the genus *Endomicrobiellum* and putatively endosymbiotic *Alphaproteobacteria* and *Verrucomicrobiae* documents convergent evolution among unrelated intracellular symbionts of termite gut flagellates (Mies et al. 2024).

Convergent evolution is also evidenced by the acquisition of a hexuronate transporter (ExuT) and all enzymes of the 2‐keto‐3‐deoxy‐phosphogluconate (KDPG) pathway (Suvorova et al. 2011) by termite‐associated *Bifidobacteriaceae* (Figure 4). The capacity to metabolise hexuronic acids is a common trait of many intracellular symbionts of termite gut flagellates, including *Azobacteroides pseudotrichonymphae* (*Bacteroidota*) (Hongoh et al. 2008), *Ancillula trichonymphae* (Strassert et al. 2016), and *Endomicrobiellum trichonymphae* (*Elusimicrobiota*) (Zheng, Dietrich, and Brune 2017). Comparative analysis of a wide range of MAGs from termite guts revealed that the KDPG pathway is present in all members of these endosymbiotic lineages and was independently acquired from other gut bacteria, possibly as an adaptation to the products of hemicellulose digestion provided by their flagellate host (Mies et al. 2024).

### 3.4 Interaction With Flagellate Host

While the acquisition of new metabolic functions by the endosymbionts of termite gut flagellates provides access to substrates provided by the host environment, such as sugar phosphates and hexuronates, the basis for a mutualistic interaction is unclear. Although the biosynthetic capacities of termite gut flagellates are unknown, it is generally assumed that the symbionts provide their hosts with important nutrients such as cofactors and amino acids, which are scarce in the lignocellulose‐rich diet of termites. However, the termite gut itself is more nutrient‐rich, and an export of essential nutrients from the symbiont to the host remains to be documented. Moreover, functional traits that are potentially beneficial for the host are rarely common among all members of an endosymbiotic lineage.

It is therefore remarkable that the threonine/serine exporter (ThrE) of *Actinomycetales* is conserved in the genera *Ancillula* and *Opitulatrix* (Figures 4 and 5). ThrE is an active transporter that was first discovered in *Corynebacterium glutamicum* , where it serves to avoid the intracellular accumulation of threonine and structurally related solutes (Simic, Sahm, and Eggeling 2001). Homologues are present in most *Bifidobacteriaceae* and many other members of the order *Actinomycetales* (Figure A8). ThrE is the first amino acid exporter identified in the genome of a flagellate endosymbiont and may provide the basis for a nutritional symbiosis at least between members of the genus *Ancillula* and their flagellate hosts. In the case of the genus *Opitulatrix*, the loss of the capacity for threonine and serine production, together with a much lower symbiont load (Table S5), suggests that formerly mutualistic symbionts have lost their beneficial role due to progressive genome erosion. They may eventually disappear from their flagellate hosts because they no longer provide a selective advantage. A similar case exists in certain *Endomicrobiellum* lineages, which compensated for severe gene losses in energy metabolism with the acquisition of an ATP/ADP antiporter (Mies et al. 2024). However, direct evidence for an exchange of metabolites between gut flagellates and their bacterial symbionts is so far lacking.

Another potentially beneficial role of termite‐associated *Bifidobacteriaceae* is the supplementation of essential cofactors required by their flagellate hosts. *Bifidobacteriaceae* colonising the mammalian gut are avid producers of various B vitamins (LeBlanc et al. 2013), and the provision of B vitamins is a common trait among intracellular symbionts of insect tissues (Blow et al. 2020; Michalik et al. 2023). Nothing is known about the biosynthetic capacities of termite gut flagellates, but since most termite‐associated *Bifidobacteriaceae* have lost a significant amount of their biosynthetic potential for vitamin production (Figure 5), the provision of B vitamins is an unlikely basis for the symbiosis.

*Bifidobacteriaceae* are also common in the intestines of honeybees, bumblebees and wasps (Kopecný et al. 2010). The bifidobacterial species in these insects represent a different lineage (Bifidobacterium+Bombiscardovia) from those found in cockroaches and termites (Figure 6), suggesting that these symbionts were acquired independently at least three times by different host lineages. *Bifidobacterium* spp. that colonise the guts of honey bees have been attributed to protective functions against bacterial pathogens (Forsgren et al. 2010; Vásquez et al. 2012). Similar to the bifidobacterial lineages found in mammals, they also influence the production of developmental hormones in the host insect (Kešnerová et al. 2017) and may play a role in hemicellulose degradation by producing various carbohydrate‐active enzymes (Bottacini et al. 2012; Kwong and Moran 2016). The loss of these functions in termite‐associated *Bifidobacteriaceae* is most likely an adaptation to their intracellular environment within the flagellate host.

### 3.5 Taxonomy

The delineation of the family *Bifidobacteriaceae* is based on phylogenetic criteria and supported by biochemical characteristics such as the presence of fructose‐6‐phosphate phosphoketolase activity (Biavati 2015; Stackebrandt, Rainey, and Ward‐Rainey 1997). Based on 16S rRNA gene phylogenies reconstructed with distance‐based methods, where *Bifidobacteriaceae* occupied a sister position to *Actinomycetales*, the family was classified in the order *Bifidobacteriales* (Stackebrandt, Rainey, and Ward‐Rainey 1997; Zhi, Li, and Stackebrandt 2009). However, phylogenomic analyses using concatenated gene trees place *Bifidobacteriaceae* within the radiation of *Actinomycetales* (GTDB, Figure 1), rendering the latter polyphyletic. Therefore, we propose to transfer the family *Bifidobacteriaceae* to the order *Actinomycetales* and to delete the now vacant order *Bifidobacteriales* from the class *Actinomycetia* under the rules of the *International Code of Nomenclature of Prokaryotes* (ICNP, Oren et al. 2023). An emended description of the family *Bifidobacteriaceae* is given below.

The current GTDB taxonomy (release 220) recognises the genomes of termite‐associated *Bifidobacteriaceae* as four genus‐level lineages of the family *Bifidobacteriaceae*, which is in agreement with our phylogenomic analysis and the RED values of the internal nodes (Figure 1). Further support for their inclusion in the family include the synapomorphies shared with other family members, such as loss of pyruvate dehydrogenase and aerobic metabolism, and the metabolism of hexoses via the fructose‐6‐phosphate phosphoketolase pathway.

The presence of both high‐quality genomes and 16S rRNA gene sequences for most lineages allows us to propose new taxa under the Code of Nomenclature of Prokaryotes Described from Sequence Data (SeqCode) (Hedlund et al. 2022; Whitman et al. 2022). The names of the new genera and species are listed in Table 1, along with the designated nomenclatural types. Moreover, we propose to remove the order of *Bifidobacteriales* and transfer the family *Bifidobacteriaceae* to the order *Actinomycetales*. The protologues including etymologies and the full description of all taxa can be found in appendix (Text B).

**TABLE 1:** New taxa of termite‐associated *Bifidobacteriaceae* proposed under SeqCode and the designated taxonomic types. The full protologues are in appendix (Text B).

New or emended taxa	Taxonomic type (genome acc. no.)
Family	
*Bifidobacteriaceae* Stackebrandt, Rainey, and Ward‐Rainey 1997 emend. Kästle Silva and Brune	Bifidobacterium bifidum
Genus	
*Ancillula* gen. nov. Strassert and Brune	*Ancillula trichonymphae* sp. nov.
*Nutricula* gen. nov. Kästle Silva and Brune	*Nutricula glyptotermitis* sp. nov.
*Opitulatrix* gen. nov. Kästle Silva and Brune	*Opitulatrix roisinitermitis* sp. nov.
*Servula* gen. nov. Kästle Silva and Brune	*Servula neotermitis* sp. nov.
Species	
*Ancillula trichonymphae* sp. nov. Strassert and Brune	GCA_000485475.1
*Ancillula hodotermitis* sp. nov. Kästle Silva and Brune	GCA_031262545.1
*Ancillula glyptotermitis* sp. nov. Kästle Silva and Brune	GCA_031268675.1
*Ancillula kalotermitis* sp. nov. Kästle Silva and Brune	GCA_031273495.1
*Ancillula prorhinotermitis* sp. nov. Kästle Silva and Brune	GCA_031280795.1
*Nutricula glyptotermitis* sp. nov. Kästle Silva and Brune	GCA_031263495.1
*Opitulatrix roisinitermitis* sp. nov. Kästle Silva and Brune	GCA_031288355.1
*Opitulatrix cubana* sp. nov. Kästle Silva and Brune	GCA_031283665.1
*Servula neotermitis* sp. nov. Kästle Silva and Brune	GCA_031281695.1

#### 3.5.1 Emended Description of *Bifidobacteriaceae* Stackebrandt, Rainey, and Ward‐Rainey 1997

Family defined by phylogenomic analysis. Members form a monophyletic group that shows a relative evolutionary divergence (RED) similar to that of the neighbouring families. Contains the genera ‘*Ancillula*’ Strassert and Brune, *Aeriscardovia* Simpson et al. 2004, *Alloscardovia* Huys et al. 2007, *Bifidobacterium* Orla‐Jensen 1924, *Bombiscardovia* Killer et al. 2010, *Galliscardovia* Pechar et al. 2017, *Gardnerella* Greenwood and Pickett 1980, *Metascardovia* Okamoto et al. 2007, *Neoscardovia* García‐Aljaro et al. 2012, ‘*Nutricula*’ Kästle Silva and Brune, ‘*Opitulatrix*’ Kästle Silva and Brune, *Parascardovia* Jian and Dong 2002, *Pseudoscardovia* Killer et al. 2013, *Scardovia* Jian and Dong 2002, and ‘*Servula*’ Kästle Silva and Brune.

Type genus: *Bifidobacterium* Orla‐Jensen 1924 (Approved Lists 1980).

Parent taxon: *Actinomycetales* Buchanan 1917 (Approved Lists 1980).

### 3.6 Conclusion

Termite‐associated *Bifidobacteriaceae* are the first representatives of *Actinomycetota* that occur intracellularly in protists. They are found exclusively in lower termites but are highly abundant only in the genus *Incisitermes*, where members of the genus *Ancillula* colonise gut flagellates of the genus *Trichonympha*. Substantial genome erosion in all genus‐level lineages strongly suggests that also the other genera are intracellular symbionts, but their specific hosts remain elusive. A genus‐level lineage of putatively free‐living *Bifidobacteriaceae* detected in cockroach guts is only distantly related. The conservation of numerous biosynthetic pathways in the genus *Ancillula* and a threonine/serine exporter in most lineages provides the first direct evidence for the long‐standing hypothesis that the intracellular symbionts of termite gut flagellates provide their hosts with amino acids. In other genera, however, many of the metabolic capacities were lost, suggesting that the originally mutualistic symbiosis is on its decline. Our study strongly supports the hypothesis that the evolution of flagellate symbionts is influenced by both progressive genome erosion and the acquisition of new functions. These new functions not only compensate for gene losses but also provide convergent adaptations to the intracellular lifestyle through horizontal gene transfer from other endosymbionts.

## 4 Experimental Procedures

### 4.1 Annotation of Metagenome‐Assembled Genomes

The MAGs of termite‐associated *Bifidobacteriaceae* were obtained in the course of a metagenomic study of numerous lower and higher termites (Mies et al. 2024). For a detailed list of MAGs and reference genomes included in the present study, see Table S2. Completeness and contamination of MAGs were assessed using CheckM (Parks et al. 2015; Chklovski et al. 2023). The absence of four single‐copy genes in the lineage‐specific marker set of CheckM v.1. from all genomes of *Bifidobacteriaceae*, including the circularised genomes of all type species (Table S6), caused a considerable bias in the completeness values. The situation did not improve when CheckM v.2 was used, which is based on a reference‐independent machine‐learning algorithm. Therefore, we removed the offending genes from the set of marker genes used by CheckM v.1. MAGs with > 90% completeness and < 5% contamination were considered high‐quality (HQ) genomes, those with > 50% completeness and < 10% contamination medium‐quality (MQ) genomes.

### 4.2 Genome Annotation and Phylogenomic Analysis

The GTDB reference database (r.220) (Parks et al. 2022) and the accompanying GTDB‐Toolkit (v. 2.4) (Chaumeil et al. 2022) standard pipeline for the classification of MAGs and genomes were used to align and classify our MAGs together with reference *Actinomycetales* and *Bifidobacteriaceae* genomes. The toolkit uses both the average nucleotide identity (ANI) and the RED scores to infer taxonomic classification. The multiple sequence alignment (MSA) output based on 120 bacterial marker genes was used to reconstruct a maximum‐likelihood genome tree in IQ‐TREE (Minh et al. 2020). Trees were inferred using the best model of evolution recommended by Model Finder and using UFBoot (Hoang et al. 2018) and the Shimodaira–Hasegawa approximate‐likelihood ratio test (SH‐aLRT) (Guindon et al. 2010) for node support assessment.

Genes were predicted and annotated using Prokka (Seemann 2014). Ghost koala (Kegg) (Kanehisa, Sato, and Morishima 2016) was used for automated MAGs annotation, and SignalP 6.0 (Teufel et al. 2022) software was used for signal peptide prediction in the identification of secreted proteins and transport systems. Since the termite‐associated *Bifidobacteriaceae* represent a novel lineage poorly represented in public databases, the annotation of the MAGs was refined by Hidden Markov Model (HMM) searching in HMMER version 3.3 (Finn, Clements, and Eddy 2011) using hmmsearch (Eddy 2011) and HMMs from Pfam (release 33.1) (El‐Gebali et al. 2019) and TIGRFAM (Haft, Selengut, and White 2003) (release 15.0) databases against a database containing annotated termite gut MAGs as well as curated sequences from SwissProt (The UniProt Consortium 2023). Results were imported for visualisation into iTOL (v.3) (Letunic and Bork 2016). Identification of pseudogenes is based on the automatic annotation of NCBI PGAP (Li et al. 2020). Clustered regularly interspaced palindromic repeats (CRISPR) arrays and their associated cas proteins were searched for in the MAGs using CRISPRCasFinder (Couvin et al. 2018). Transposable elements were predicted in the MAGs using Issaga (Varani et al. 2011).

Additionally, antiSMASH 7.1 (Blin et al. 2023) was used for the annotation and analysis of secondary metabolite biosynthetic gene clusters. For the annotation and classification of carbohydrate‐active enzymes, the MAGs were submitted to dbCAN3 (Zheng et al. 2023). Hydrogenases were classified with the HydDB database (Søndergaard, Pedersen, and Greening 2016).

To create the protein phylogenies, we gathered amino acid sequences from the termite‐associated *Bifidobacteriaceae* MAGs. Homologues were then extracted from a custom database containing termite gut MAGs and non‐redundant high‐quality SWISS‐PROT entries using BlastP (v. 2.6.0). These sequences were aligned using MAFFT (v. 7.427) and used to build a tree with IQ‐TREE, using the best model of evolution recommended by Model Finder (Kalyaanamoorthy et al. 2017; Minh et al. 2020). Node support was assessed using SH‐aLRT (Guindon et al. 2010) and ultrafast bootstrap analysis (UFBoot; Hoang et al. 2018). The alignment and consensus tree were then imported into Arb software (v. 6.1) (Ludwig et al. 2004), where we made manual improvements. If needed, we exported the alignment to generate a second tree using the same settings.

### 4.3 16S rRNA‐Based Diversity

Full‐length 16S rRNA gene sequences were extracted from gut metagenomes of lower and higher termites (Table S1) and MAGs of termite‐associated *Bifidobacteriaceae* (Table S2) using Barrnap (v. 0.9) (Seemann 2013). Together with sequences previously obtained from clone libraries and long‐read amplicon libraries (Table S4), they were imported into the ARB‐SILVA database (v. 138.1) (Quast et al. 2013) using the ARB software package (v. 7.02) (Ludwig et al. 2004), aligned with SINA (v1.2.12) (Pruesse, Peplies, and Glöckner 2012), and placed into the phylogenetic framework of Silva v.138.1. The overall alignment was manually curated using the alignment editor integrated into ARB (Protasov et al. 2023). The improved alignment was exported and a maximum‐likelihood tree was inferred with IQ‐TREE using the substitution model suggested by ModelFinder. Node support was assessed with SH‐aLRT and UFBoot.

### 4.4 Fluorescence In Situ Hybridization

*Incisitermes incisus* and *Incisitermes tabogae* (Gualedoupe), *Glyptotermes* species 5 (Cameroon), and *Kalotermes italicus* (Italy) were collected in the field. *Kalotermes flavicollis* was obtained from the Bundesanstalt für Materialforschung (BAM) in Berlin, Germany. The FISH followed a previously published protocol, using gut contents of live specimens fixed with paraformaldehyde (Strassert et al. 2012). Termite‐associated *Bifidobacteriaceae* and other bacteria were visualised by double hybridization with the previously established oligonucleotide probe ACT490 (5′‐AGCCGGTCCTTTCTTGTTAG‐3′; Strassert et al. 2012) and a general *Bacteria*‐specific probe (EUB338); non‐specific probe binding was checked by additional hybridizations using a nonsense probe (NON338EUB; Wallner, Amann, and Beisker 1993).

## Author Contributions

**Joana Kästle Silva:** conceptualization, methodology, data curation, investigation, validation, formal analysis, supervision, visualization, writing – original draft, project administration, writing – review and editing. **Vincent Hervé:** data curation, formal analysis, resources, writing – review and editing. **Undine S. Mies:** data curation, resources. **Katja Platt:** resources, writing – review and editing, methodology. **Andreas Brune:** writing – original draft, writing – review and editing, visualization, supervision, resources, funding acquisition, conceptualization, methodology, validation, data curation, project administration, formal analysis, investigation.

## Conflicts of Interest

The authors declare no conflicts of interest.

## Supporting information


Data S1.

## Figures and Tables

**FIGURE A12:**
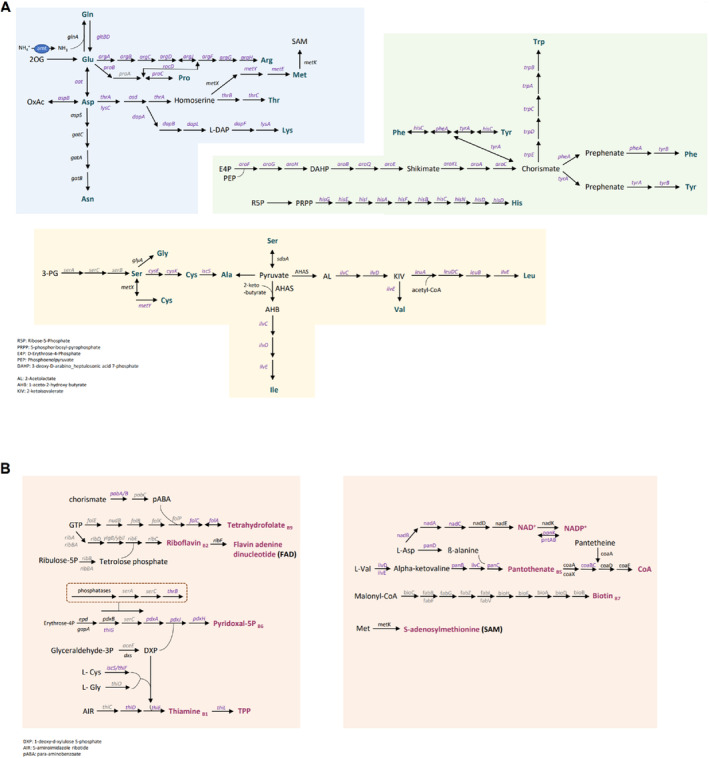
Expanded biosynthetic pathways for amino acid (A) and cofactor (B) biosynthesis in the *Ancillula* and *Opitulatrix* MAGs. Genes in black are encoded by both genera. Genes in purple are present only in *Ancillula*, and genes in grey are absent in both genera.

## Data Availability

The metagenomic datasets used in this study are available in the NCBI Sequence Read Archive (SRA) (Table S1). The GenBank accession numbers of the MAGs are listed in Table S2. For the GenBank accession numbers of the 16S rRNA gene sequences obtained in this study¸ see Tables S1, S2 and S4. The Supporting information, including tables, can be accessed using the following DOI: 10.5281/zenodo.14186872.

## Appendix B

### Protologues for New Prokaryotic Taxa

Etymology and description of the new taxa proposed under SeqCode.

### *Bifidobacteriaceae* Stackebrandt, Rainey, and Ward‐Rainey 1997 emend. Kästle Silva and Brune

Family defined by phylogenomic analysis. Members form a monophyletic group that shows a relative evolutionary divergence (RED) similar to that of the neighbouring families. Contains the genera *Ancillula* Strassert and Brune, *Aeriscardovia* Simpson et al. 2004, *Alloscardovia* Huys et al. 2007, *Bifidobacterium* Orla‐Jensen 1924, *Bombiscardovia* Killer et al. 2010, *Galliscardovia* Pechar et al. 2017, *Gardnerella* Greenwood and Pickett 1980, *Metascardovia* Okamoto et al. 2007, *Neoscardovia* García‐Aljaro et al. 2012, *Nutricula* Kästle Silva and Brune, *Opitulatrix* Kästle Silva and Brune, *Parascardovia* Jian and Dong 2002, *Pseudoscardovia* Killer et al. 2013, *Scardovia* Jian and Dong 2002, and *Servula* Kästle Silva and Brune.

### *Ancillula* gen. nov. Strassert and Brune

*Etymology*: An.cilˈlu.la. L. fem. dim. n. *Ancillula*, a little female servant.

*Synonym*: ‘*Candidatus* Ancillula’ Strassert et al. 2012 (not validly published).

*Description*: Genus defined by phylogenomic analysis of metagenome‐assembled genomes and single‐cell amplified genomes. Members of the genus form a monophyletic group that shows a relative evolutionary divergence (RED) similar to that of the neighbouring genera.

*Type species*: *Ancillula trichonymphae* sp. nov.

#### *Ancillula trichonymphae* sp. nov. Strassert and Brune

*Etymology*: tri.cho.nymˈphae. N.L. gen. fem. n. *trichonymphae*, of *Trichonympha*, a genus of termite gut flagellates.

*Synonym*: ‘*Candidatus* Ancillula trichonymphae’ Strassert et al. 2012 (not validly published).

*Description*: Short, slightly pleomorphic rods (approximately 1.2 mm in length and 0.3 mm in diameter) with a typical Gram‐positive cell wall. Colonise the cytoplasm of a lineage of closely related flagellates (Trichonympha Cluster II) in the hindgut of termites. SSU rRNA genes form a cluster of host‐specific phylotypes.

*Type genome*: ImTpAt1^ST^, GCA_000485475.1 (genome); JQ993633 (16S rRNA).

*Additional genomes*: GCA_031276145.1.

#### *Ancillula hodotermitis* sp. nov. Kästle Silva and Brune

*Etymology*: ho.do.ter'mi.tis. N.L. gen. n. *hodotermitis*, of *Hodotermes*, a genus of termites.

*Description*: The species comprises one metagenome‐assembled genome. The species will include all bacteria with more than 95% average nucleotide identity (ANI) to the type genome. The GC content of the type genome is 34.5% and the estimated genome size is 1.51 Mbp.

*Type genome*: Hm464_bin179, GCA_031262545.1 (genome), OR396865 (16S rRNA).

#### *Ancillula glyptotermitis* sp. nov. Kästle Silva and Brune

*Etymology*: glyp.to.terˈmi.tis. N.L. gen. n. *glyptotermitis*, of *Glyptotermes*, a genus of termites.

*Description*: The species comprises one metagenome‐assembled genome. The species will include all bacteria with more than 95% average nucleotide identity (ANI) to the type genome. The GC content of the type genome is 37.92% and the estimated genome size is 1.80 Mbp.

*Type genome*: Gsp477_bin129 from the lower termite *Glyptotermes*.

*GenBank accession number*: GCA_031268675.1 (genome), OR396855 (16S rRNA).

#### *Ancillula kalotermitis* sp. nov. Kästle Silva and Brune

*Etymology*: ka.lo.ter'mi.tis. N.L. gen. n. *kalotermitis*, of *Kalotermes*, a genus of termites.

*Description*: The species comprises one metagenome‐assembled genome. The species will include all bacteria with more than 95% average nucleotide identity (ANI) to the type genome. The GC content of the type genome is 49.22% and the estimated genome size is 1.07 Mbp.

*Type genome*: Kf353_bin77 from the lower termite *Kalotermes flavicollis*.

*GenBank accession number*: GCA_031273495.1 (genome), OR396858 (16S rRNA).

#### *Ancillula prorhinotermitis* sp. nov. Kästle Silva and Brune

*Etymology*: pro.rhi.no.terˈmi.tis. N.L. gen. n. *prorhinotermitis*, of *Prorhinotermes*, a genus of termites.

*Description*: The species comprises one metagenome‐assembled genome. The species will include all bacteria with more than 95% average nucleotide identity (ANI) to the type genome. The GC content of the type genome is 46.36% and the estimated genome size is 0.673 Mbp.

*Type genome*: Pc512_bin108 from the lower termite *Prorhinotermes canalifrons*.

*GenBank accession number*: GCA_031280795.1 (genome), PP623112 (16S rRNA).

### *Opitulatrix* gen. nov. Kästle Silva and Brune

*Etymology*: O.pi.tu.laˈtrix. N.L. fem. n. *Opitulatrix*, a female helper, maid.

*Description*: Genus defined by phylogenomic analysis of metagenome‐assembled genomes. Members of the genus form a monophyletic group that shows a relative evolutionary divergence (RED) similar to that of the neighbouring genera.

*Type species*: *Opitulatrix roisinitermitis* sp. nov.

#### *Opitulatrix roisinitermitis* sp. nov. Kästle Silva and Brune

*Etymology*: roi.si.ni.termi.tis. N.L. gen. n. *roisinitermitis*, of *Roisinitermes*, a genus of termites.

*Description*: The species comprises only metagenome‐assembled genomes. The species includes all bacteria with more than 95% average nucleotide identity (ANI) to the type genome. The GC content of the type genome is 33.45% and the estimated genome size is 0.98 Mbp.

*Type genome*: Roe453_bin28 from the lower termite *Roisinitermes ebogoensis*.

*GenBank accession number*: GCA_031288355.1 (genome); OR396864 (16S rRNA).

*Additional genomes*: GCA_031288055.1, GCA_031288095.1.

#### *Opitulatrix cubana* sp. nov. Kästle Silva and Brune

*Etymology*: cu.ba'na. N.L. fem. adj. cubana, of Cuba, Cuban, referring to the geographic origin of the host.

*Description*: The species comprises one metagenome‐assembled genomes. The species will include all bacteria with more than 95% average nucleotide identity (ANI) to the type genome. The GC content of the type genome is 35.44% and the estimated genome size is 0.768 Mbp.

*Type genome*: Ncb351_bin81 from the lower termite *Neotermes cubanus*.

*GenBank accession number*: GCA_031283665.1 (genome); OR396859 (16S rRNA).

### *Nutricula* gen. nov. Kästle Silva and Brune

*Etymology*: Nu.triˈcu.la. L. fem. n. *nutricula*, a wet nurse.

*Description*: Genus defined by phylogenomic analysis of metagenome‐assembled genomes. The single genome shows a relative evolutionary divergence (RED) similar to that of the neighbouring genera.

*Type species*: *Nutricula glyptotermitis* sp. nov.

#### *Nutricula glyptotermitis* sp. nov. Kästle Silva and Brune

*Etymology*: glyp.to.ter'mi.tis. N.L. gen. n. *glyptotermitis*, of *Glyptotermes*, a genus of termites.

*Description*: The species comprises one metagenome‐assembled genome. The species will include all bacteria with more than 95% average nucleotide identity (ANI) to the type genome. The GC content of the type genome is 43.55% and the estimated genome size is 1.54 Mbp.

*Type genome*: Gx485_bin43 from the lower termite *Glyptotermes* sp.

*GenBank accession number*: GCA_031263495.1 (genome); OR396856 (16S rRNA).

### *Servula* gen. nov. Kästle Silva and Brune

*Etymology*: Serˈvu.la. L. fem. dim. n. *servula*, a young servant girl.

*Description*: Genus defined by phylogenomic analysis of metagenome‐assembled genomes. The single genome shows a relative evolutionary divergence (RED) similar to that of the neighbouring genera.

*Type species*: *Servula neotermitis* sp. nov.

#### *Servula neotermitis* sp. nov. Kästle Silva and Brune

*Etymology*: ne.o.termi.tis. N.L. gen. n. *neotermitis*, of *Neotermes*, a genus of termites.

*Description*: The species comprises one metagenome‐assembled genome. The species will include all bacteria with more than 95% average nucleotide identity (ANI) to the type genome. The GC content of the type genome is 42.03% and the estimated genome size is 1.10 Mbp.

*Type genome*: Nm470_bin85 from the lower termite *Neotermes meruensis*.

*GenBank accession number*: GCA_031281695.1 (genome); OR396861 (16S rRNA).
